# Induction of RAGE Shedding by Activation of G Protein-Coupled Receptors

**DOI:** 10.1371/journal.pone.0041823

**Published:** 2012-07-30

**Authors:** Verena V. Metz, Elzbieta Kojro, Dorothea Rat, Rolf Postina

**Affiliations:** Institute of Pharmacy and Biochemistry, Johannes Gutenberg University, Mainz, Germany; Duke University, United States of America

## Abstract

The multiligand Receptor for Advanced Glycation End products (RAGE) is involved in various pathophysiological processes, including diabetic inflammatory conditions and Alzheime
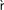
s disease. Full-length RAGE, a cell surface-located type I membrane protein, can proteolytically be converted by metalloproteinases ADAM10 and MMP9 into a soluble RAGE form. Moreover, administration of recombinant soluble RAGE suppresses activation of cell surface-located RAGE by trapping RAGE ligands. Therefore stimulation of RAGE shedding might have a therapeutic value regarding inflammatory diseases. We aimed to investigate whether RAGE shedding is inducible via ligand-induced activation of G protein-coupled receptors (GPCRs). We chose three different GPCRs coupled to distinct signaling cascades: the V2 vasopressin receptor (V2R) activating adenylyl cyclase, the oxytocin receptor (OTR) linked to phospholipase Cβ, and the PACAP receptor (subtype PAC1) coupled to adenylyl cyclase, phospholipase Cβ, calcium signaling and MAP kinases. We generated HEK cell lines stably coexpressing an individual GPCR and full-length RAGE and then investigated GPCR ligand-induced activation of RAGE shedding. We found metalloproteinase-mediated RAGE shedding on the cell surface to be inducible via ligand-specific activation of all analyzed GPCRs. By using specific inhibitors we have identified Ca^2+^ signaling, PKCα/PKCβI, CaMKII, PI3 kinases and MAP kinases to be involved in PAC1 receptor-induced RAGE shedding. We detected an induction of calcium signaling in all our cell lines coexpressing RAGE and different GPCRs after agonist treatment. However, we did not disclose a contribution of adenylyl cyclase in RAGE shedding induction. Furthermore, by using a selective metalloproteinase inhibitor and siRNA-mediated knock-down approaches, we show that ADAM10 and/or MMP9 are playing important roles in constitutive and PACAP-induced RAGE shedding. We also found that treatment of mice with PACAP increases the amount of soluble RAGE in the mouse lung. Our findings suggest that pharmacological stimulation of RAGE shedding might open alternative treatment strategies for Alzheimeŕs disease and diabetes-induced inflammation.

## Introduction

The Receptor for Advanced Glycation End products (RAGE) is a type I transmembrane protein belonging to the immunoglobulin superfamily and is usually expressed at low levels in epithelial, neuronal and vascular cells. The lung is the sole organ having high expression of RAGE under normal conditions [1].

RAGE has been shown to play a crucial role in chronic inflammatory diseases, late diabetic complications, atherosclerosis and Alzheimeŕs disease [2]. Proteins and peptides such as advanced glycation end products (AGEs), Aβ peptides, S100/calgranulin family members and HMGB1 (amphoterin, high-mobility group protein B1) have been identified as ligands for RAGE [3]. Ligand binding of RAGE induces production of proinflammatory cytokines from macrophages [4], [5] and amplifies inflammatory responses [6]. Moreover, the expression of RAGE is induced by an autocrine mechanism upon the binding of RAGE ligands [7]. The concentration of AGEs is enhanced under some pathological circumstances such as diabetes mellitus, inflammation, oxidative stress, renal failure [8] and Alzheimeŕs disease [9]. Therefore, in these pathological conditions the ligand-induced increase of full-length RAGE expression contributes to the severity of these diseases.

Numerous studies have shown that administration of soluble RAGE (sRAGE) can alleviate full-length RAGE-mediated harmful processes by trapping RAGE ligands and preventing RAGE signaling. For example the application of sRAGE slowed-down tumor growth and reduced the amount of metastases in mice [10]. Other studies demonstrate that treatment with sRAGE can completely suppress diabetic atherosclerosis [11] and reverse vascular hyperpermeability in diabetic rats [12]. Injection of soluble RAGE into the brain of an Alzheimeŕs disease mouse model reduced the levels of Aβ, Aβ plaques and BACE1 (beta-site APP Cleaving Enzyme 1) [13]. We as well as others have shown that full-length RAGE is subjected to protein ectodomain shedding conducted by metalloproteinase ADAM10 [14], [15], [16]. ADAM10 (A Disintegrin And Metalloproteinase 10) is a multidomain type I transmembrane zinc-dependent metalloproteinase [17]. Shedding processes are known to be inducible by calcium ionophores and phorbol esters. Moreover α-secretase-mediated shedding of the amyloid precursor protein (APP) is achievable by ligand-induced activation of G protein-coupled receptors (GPCRs) [18], [19], [20].

As soluble RAGE alleviates pathophysiological processes mediated by full-length RAGE, the stimulation of RAGE shedding may be used as a therapeutic attempt in the treatment of diseases such as Alzheimer and diabetes mellitus. The aim of our study was to investigate whether full-length RAGE is proteolytically converted into soluble RAGE following activation of G protein-coupled receptors (GPCRs). To answer this question, we investigated GPCRs stimulating various main signaling networks: the V2 vasopressin coupled to adenlylyl cyclase [21], the oxytocin receptor linked to phospholipase Cβ [22] and the PAC1 (pituitary adenylate cyclase-activating polypeptide) receptor known to be able to activate adenylyl cyclase, phospholipase Cβ, calcium signaling and MAP (mitogen-activated protein) kinases [23].

The neuropeptide PACAP exhibits anti-inflammatory and neuroprotective properties primarily mediated through the PAC1 receptor [23]. Moreover, in previous studies we demonstrated that activation of the PAC1 receptor induces α-secretase ADAM10-mediated APP cleavage in cultured cells [19] and *in vivo* [24]. Thus, the PACAP/PAC1 system provides an ideal model to investigate signaling pathways involved in metalloproteinase-induced RAGE shedding. Since PACAP receptors are also present in the lung, being the main source of endogenous RAGE, we analyzed lung samples of mice treated with the PACAP-38 peptide for 3 months.

## Results

### The Shedding of RAGE is Inducible by Activation of Different G Protein-coupled Receptors

**Figure 1:**
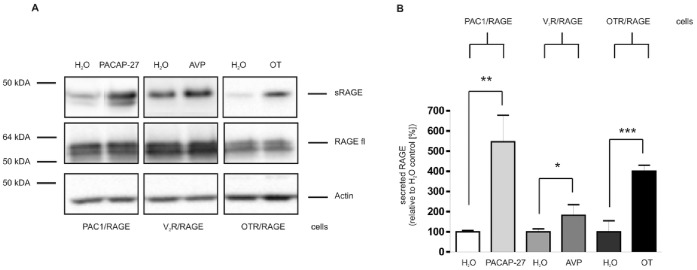
Stimulation of RAGE shedding via ligand-induced activation of different G protein-coupled receptors in HEK cells. The cell line co-expressing RAGE and the PAC1 receptor (PAC1/RAGE) was stimulated with PACAP-27, cells co-expressing RAGE and the V2 vasopressin receptor (V2R/RAGE) were treated with arginine-vasopressin (AVP) and cells co-expressing RAGE and the oxytocin receptor (OTR/RAGE) were stimulated with oxytocin (OT). As control, cell lines were mock-stimulated with comparable volumes of water. After stimulation with 300 nM of each hormone for 4 h the cell culture supernatant was collected and the proteins were precipitated. Secreted RAGE (sRAGE) was detected by Western blotting using the polyclonal antibody 3260, followed by an anti-rabbit antibody labeled with horseradish peroxidase and ECL. Full-length RAGE and actin were detected in cell lysates by Western blot analysis. For quantification the experiments were done in triplicates. (A) Western Blots for secreted RAGE (sRAGE) in cell culture supernatants, full-length RAGE (RAGE fl) and Actin in cell lysates. (B) Quantitative analysis: Shown are the mean effects ± S.D., significance was determined by the One-way ANOVA Bonferroni test (*  =  P<0.05; **  = 0.01; ***  =  P<0.001).

For investigation whether the proteolytic machinery responsible for the shedding of RAGE is inducible via activation of G protein-coupled receptors, we generated cell lines co-expressing RAGE and a respective GPCR. The PAC1 receptor, the V2 vasopressin and the oxytocin receptor were included in the study because these GPCRs act through different major signaling pathways. In all three cases we found the shedding of RAGE to be inducible by agonist-induced GPCR activation (Figure 1). In culture supernatant of cells challenged with respective agonist the amount of soluble RAGE was significantly increased. Whereas arginine vasopressin (AVP) treatment doubled the amount of sRAGE, PACAP and oxytocin (OT) treatment induced the shedding of RAGE 4 to 5-fold. The doublet band of sRAGE in the supernatant of PAC1/RAGE cells represents glycosylated and, with lower molecular weight, unglycosylated soluble RAGE [16]. Due to lower RAGE expression, only glycosylated RAGE is shedded from the other cell lines. The amount of full-length RAGE in cell lysates was unaltered following shedding induction (Figure 1A). For identification of the signaling pathways involved in GPCR-induced RAGE shedding we focused on the PAC1 receptor because this receptor uses a multi-branched signaling network which also covers the signaling routes usually used by the V2R and OTR. While the PAC1 receptor is known to be coupled to adenylyl cyclase, phospholipase Cβ, calcium signaling and MAP kinases, the OTR is mainly linked to phospholipase Cβ and calcium signaling and the V2R to adenylyl cyclase.

**Figure 2:**
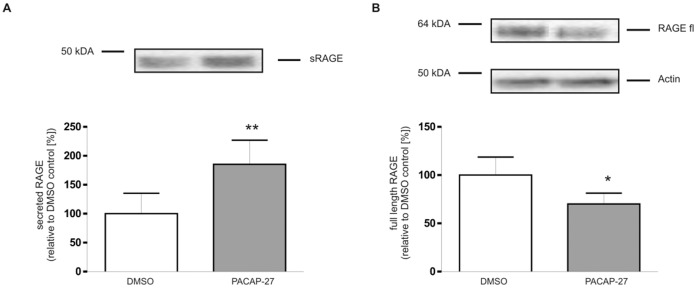
Induction of RAGE shedding at the cell surface with PACAP-27. Cell-surface proteins of full-length RAGE and PAC1 coexpressing cells (PAC1/RAGE cells) were biotinylated using a membrane-impermeable biotinylation reagent. PACAP-27 and control treatment was then performed for 2 h. The amount of secreted biotinylated (A) and cell-anchored biotinylated RAGE (B) was determined separately under constitutive or PACAP-stimulated conditions after isolation of biotinylated proteins. RAGE was detected by Western blotting using antibody Mab5328 and ECL; typical blots are shown. Left image, amount of secreted biotinylated RAGE; right images, cell surface-located biotinylated RAGE and actin in total cell lysates. For quantification the experiments were performed in triplicates. Shown are the mean effects ± S.D., significance was determined by the One-way ANOVA Bonferroni test (*  =  P<0.05; **  =  P<0.01).

Since ectodomain shedding is taking place at the cell surface, we quantified the amount of RAGE at that cellular compartment. For this purpose, cell surface proteins were labeled with a membrane-impermeable biotinylation reagent and after that shedding was induced for 2 hours with PACAP-27. Biotinylated proteins in cell culture supernatants and intact cells were isolated separately. Biotinylated secreted and full-length RAGE variants were quantified by Western blot analysis. In these experiments, PACAP-27 treatment increased the amount of secreted RAGE in the cell culture supernatant two-fold (Figure 2A) and significantly decreased the amount of plasma membrane-located full-length RAGE by 30% (Figure 2B). This experiment demonstrates that soluble RAGE is not released from intracellular pools by exocytosis, instead it is generated by proteolytic cleavage of full-length RAGE at the cell surface.

### The Shedding of RAGE is Inducible by Activation of the G Protein-coupled PAC1 Receptor in a Dose-dependent Manner

**Figure 3:**
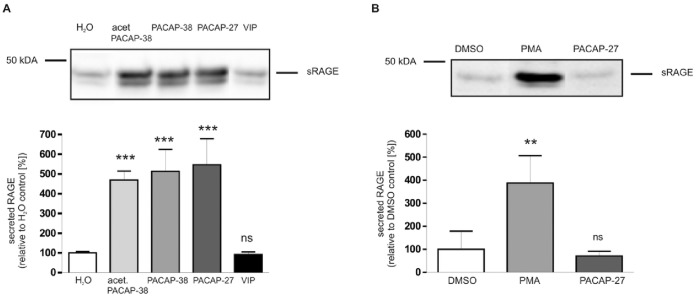
Stimulation of RAGE shedding with different PACAP peptides in PAC1/RAGE cells. (A) PAC1/RAGE cells were incubated for 4 h with either 300 nM acetylated PACAP-38, PACAP-38, PACAP-27 or VIP; H_2_O: mock-treated cells (negative control). Then sRAGE was detected and quantified by Western blotting as described in Figure 1. Shown are the mean effects ± S.D., significance was determined by the One-way ANOVA Bonferroni test (ns  =  P>0.05; ***  =  P<0.001). (B) RAGE expressing cells were incubated for 4 h with PMA (1 µM), PACAP-27 (300 nM) or DMSO as control. Detection and quantification of secreted RAGE as well as significance was determined as described in figure part A; (ns  =  P>0.05; **  =  P<0.01).

The PAC1 receptor is able to bind different PACAP ligands with similar high affinity. Treatment of PAC1/RAGE cells with 300 nM of either PACAP-27, PACAP-38 or acetylated PACAP-38 resulted in a strong and comparable induction of RAGE shedding (Figure 3A). In addition to natural PACAP peptides, acetylated PACAP-38 was used due to its increased stability against aminopeptidases. However, this did not influence the potency of shedding induction. The ligand VIP (Vasoactive Intestinal Peptide), which has a thousand-fold lower binding affinity for the PAC1 receptor than PACAP peptides (K_D_ 500 nM vs. 0.5 nM) [25], [26] was not able to induce the shedding of RAGE. To demonstrate further that the PAC1 receptor is required for the PACAP-induced stimulation of shedding, we also analyzed the effect of PACAP on HEK cells expressing only RAGE but not the PAC1 receptor. In these cells PACAP-27 was not able to induce the shedding of RAGE (Figure 3B). To validate that the shedding machinery is intact in these cells, the cells were also treated with the well-known shedding inducer PMA. Treatment with PMA (1 µM for 4 h) caused a 4-fold increase in the amount of sRAGE (Figure 3B). The phorbol ester PMA is a protein kinase C activator and a well-known shedding inducer [27] which activates RAGE shedding via calcium-dependent PKC isoforms PKCα and PKCβI [16].

Same experiments were performed on HEK/RAGE cells lacking OTR and V2R expression. On these cells, neither OT nor AVP were able to induce the release of RAGE (data not shown). Thus agonist-induction shedding of RAGE strictly requires the presence of respective GPCR.

**Figure 4:**
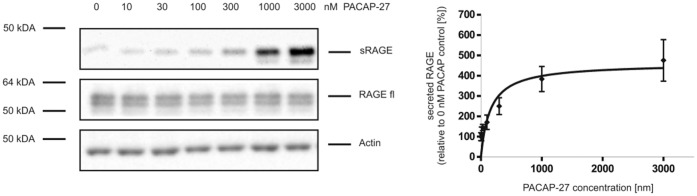
PACAP-induced dose-dependent shedding of RAGE. PAC1/RAGE cells were treated for 4 h with increasing PACAP-27 concentrations (ranging from 10 nM up to 3000 nM). Afterwards the medium was collected and sRAGE was identified with the Ab3260 as described in Figure 1. For quantification, the experiments were done in triplicates. Shown are the mean effects ± S.D. Full-length RAGE and actin were detected in total cell lysates by Western blot analysis.

Next we analyzed whether the shedding of RAGE in PAC1/RAGE cells is dependent on the PACAP concentration. Cells were treated with different PACAP-27 hormone concentrations up to 3 µM for 4 h, and then the amount of shedded RAGE was determined by Western blot analysis (Figure 4). PACAP-27 induced the shedding of RAGE in a dose-dependent and saturable manner. 300 nM PACAP-27 caused a 2.5 fold increase of soluble RAGE (sRAGE) and a 4-fold increase was detected after application of 1 µM and 3 µM PACAP-27 (Figure 4). In cell lysates, the amount of full-length RAGE (RAGE fl) was unaltered. Thus, increased amounts of sRAGE are not caused by increased RAGE expression.

**Figure 5:**
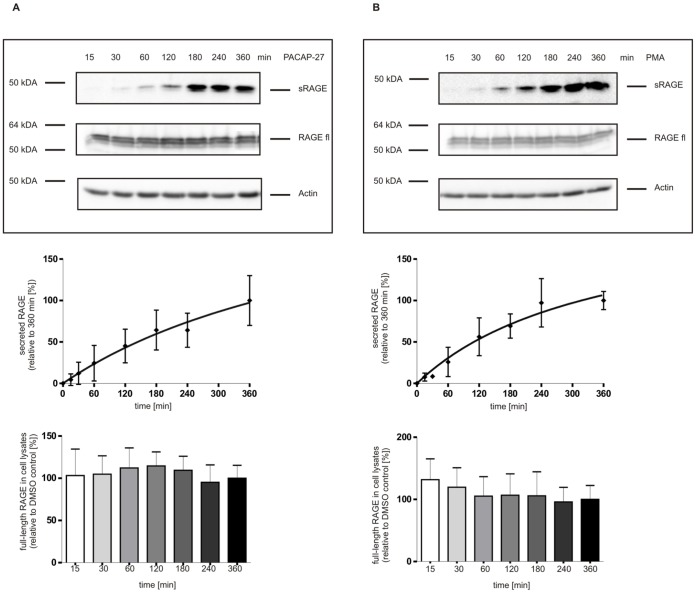
Time dependence of RAGE shedding induced by either PACAP (A) or PMA (B). Upper figure part: representative Western blots for detection of sRAGE, full-length RAGE (RAGE fl) and actin; middle figure part: Quantitative analysis of sRAGE amounts; lower figure part: Quantification of full-length RAGE expression. PAC1/RAGE cells were incubated for different periods of time (15 min up to 360 min) using either 300 nM PACAP-27 or 1 µM PMA. sRAGE was detected and quantified as described in Figure 1. Full-length RAGE and actin were analyzed in cell lysates by Western blotting using either the monoclonal anti-RAGE antibody 5328 or anti-actin antibody A2066. Detection was performed with ECL. For quantification of sRAGE and full-length RAGE, the experiments were done in triplicates. Shown are the mean effects ± S.D.

To analyze the kinetic of PACAP-induced RAGE shedding, we investigated shedding over a time period of six hours. Detectable amounts of sRAGE were noticed after 30 min of stimulation, and the amount of sRAGE continuously increased during the first three hours of 300 nM PACAP-27 treatment (Figure 5A). Longer lasting stimulation increased the amount of sRAGE only marginally and had no effect on the expression of full-length RAGE (RAGE fl). Prior to incubation with PACAP-27, cells were pre-treated with the protein biosynthesis inhibitor cycloheximide to ensure that an enhanced amount of RAGE cannot be caused by *de novo* gene expression.

To estimate the potency of PACAP-induced RAGE shedding in our cellular system, the observed effects were compared to effects of phorbol ester-mediated RAGE shedding.

Time-dependent treatment of PAC1/RAGE cells with PMA (1 µM) resulted in an increased shedding of RAGE. The kinetic of PMA-induced RAGE shedding was comparable with the PACAP-induced kinetic (Figure 5A vs. Figure 5B).

**Figure 6:**
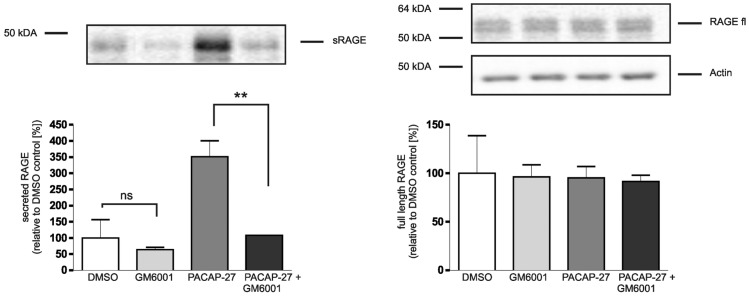
Effect of metalloproteinase inhibitor GM6001 on PACAP-induced RAGE shedding. PAC1/RAGE cells were pre-treated for 1 h with either 20 µM GM6001 or solvent, then 300 nM PACAP-27 was added for 2 h; DMSO: mock-treated cells (negative control). Secreted RAGE was detected and quantified as described in Figure 1. Full-length RAGE and actin were detected in total cell lysates by Western blot analysis. Shown are the mean effects ± S.D., significance was determined by the One-way ANOVA Bonferroni test (ns  =  P>0.05; **  =  P<0.01). Full-length RAGE and actin were detected in total cell lysates by Western blot analysis.

**Figure 7:**
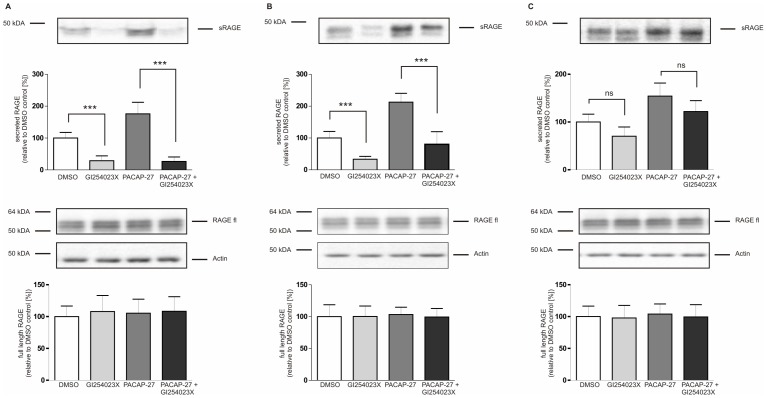
Effect of metalloproteinase inhibitor GI254023X on PACAP-induced RAGE shedding. PAC1/RAGE cells were pre-treated for 1 h with either 25 µM (A), 1 µM (B) or 100 nM (C) GI254023X or solvent (DMSO), then 300 nM PACAP-27 was added for 2 h; DMSO: mock-treated cells (negative control). Soluble RAGE was detected and quantified as described in Figure 1. Full-length RAGE and actin were detected in total cell lysates by Western blot analysis. Shown are the mean effects ± S.D., significance was determined by the One-way ANOVA Bonferroni test (ns  =  P>0.05; ***  =  P<0.001).

In a previous study we demonstrated that zinc-dependent metalloproteinases ADAM10 and MMP9 (Matrix-Metalloproteinase 9) conduct RAGE shedding [16]. Therefore, we analyzed whether PACAP-induced shedding is also mediated by metalloproteinases. Treatment of PAC1/RAGE cells with the broad spectrum metalloproteinase inhibitor GM6001 (20 µM) strongly reduced PACAP-27-mediated RAGE shedding (Figure 6). To investigate the contribution of ADAM10 and MMP9 in PACAP-induced RAGE shedding we used the ADAM10/MMP9 selective inhibitor GI254023X. In *in vitro* assays with recombinant proteinases, the IC_50_ values for ADAM10 and MMP9 are 5.3 and 2.5 nM, respectively [28]. In cellular assays, even in a high micromolar concentration (30 µM), GI254023X did only marginally inhibit PMA-induced shedding processes [28], [29]. GI254023X applied in 25 µM concentration strongly inhibited constitutive (72% inhibition) as well as the PACAP-induced shedding (86% inhibition) of RAGE (Figure 7A). At a concentration of 1 µM constitutive shedding of RAGE was reduced about 67% and PACAP-induced shedding about 75% (Figure 7B). Even at a concentration of 100 nM (Figure 7C), GI254023X slightly inhibited the shedding of RAGE, suggesting that ADAM10 and/or MMP9 are playing important roles in PACAP-induced RAGE shedding. Just like the biotinylation experiment, the data obtained with metalloproteinase inhibitors clearly demonstrate that shedding and not exocytosis is responsible for the generation of soluble RAGE.

**Figure 8:**
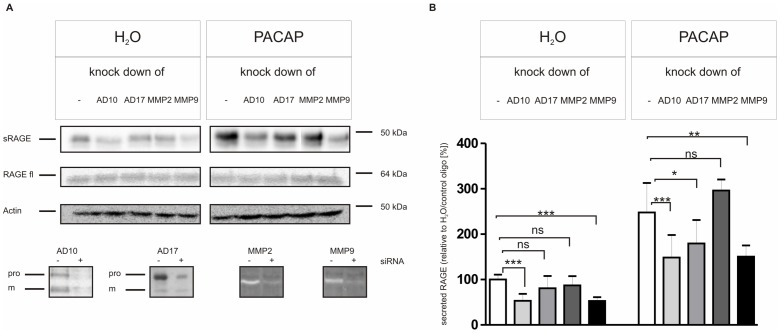
Effects of RNAi-mediated knock-down on the shedding of RAGE. PAC1/RAGE cells were transfected with stealth RNAi oligonucleotide duplexes (Invitrogen) targeting either ADAM10 (AD10), ADAM17 (AD17), MMP9 or MMP2. As control (-) cells were transfected with a stealth RNAi control oligonucleotide duplex (Invitrogen). Experiments were performed 48 h after transfection. Secretion of RAGE was analyzed under unstimulated (H_2_O, n = 10) and PACAP-induced (300 nM, n = 5) conditions for 3 h. (A) Typical Western blots and gelatin zymography are shown, (B) Quantitative analysis of secreted RAGE. RAGE secreted into the cell culture supernatant and full-length RAGE in cell lysates were detected with antibody Mab5328. The enzymatic activity of MMP9 and MMP2 in cell culture supernatants was determined by gelatin zymography as described in Materials and Methods. In cell lysates full-length RAGE (fl-RAGE), ADAM10 (pro and mature (m) forms), and ADAM17 (pro and mature (m) forms) were detected with suitable antibodies (see “Materials and Methods”). As a loading control actin was detected in cell lysates by Western blotting. Shown in the quantitative analysis (B) are the mean effects ± S.D., significance was determined by the One-way ANOVA Bonferroni test (ns  =  P>0.05; *  =  P<0.05; **  =  P<0.01; ***  =  P<0.001).

To confirm that ADAM10 and MMP9 act as sheddases on RAGE, we additionally performed siRNA-mediated knock-down experiments. Here we investigated the contribution of ADAMs 10 and 17 as well as of MMPs 2 and 9. Whereas the knock-down of ADAM10 and MMP9 resulted in a ∼ 50% reduction of constitutive RAGE shedding, the release of soluble RAGE was only slightly influenced after the knock-down of ADAM17 and not at all after knock-down of MMP2 (Figure 8). PACAP-induced RAGE shedding was mostly reduced after knock-down of ADAM10 and MMP9 expression. However, decreased PACAP-induced RAGE proteolysis was also evident after reducing ADAM17 expression. In all cases siRNA-mediated knock-downs did not prevent expression of the target protein completely and due to variations in knock-down efficiencies a comparative analysis of the contribution of metalloproteinases is impossible. Nevertheless, the result of our siRNA-mediated knock-down experiments reinforce our previous interpretation that ADAM10 and MMP9 are important players in PACAP-induced RAGE shedding. In addition, the new experiments demonstrate an involvement of ADAM17 in PACAP-induced RAGE shedding and exclude the contribution of MMP2.

### Analysis of the Signaling Pathways Involved in PACAP-induced RAGE Shedding

Binding of a PACAP agonist to the PAC1 receptor leads to activation of several intracellular signaling cascades including the adenylate cyclase system, stimulation of phospholipase C and the MAP kinase pathway [23]. Our next aim was to elucidate the signaling pathway(s) involved in PACAP-induced RAGE shedding.

**Figure 9:**
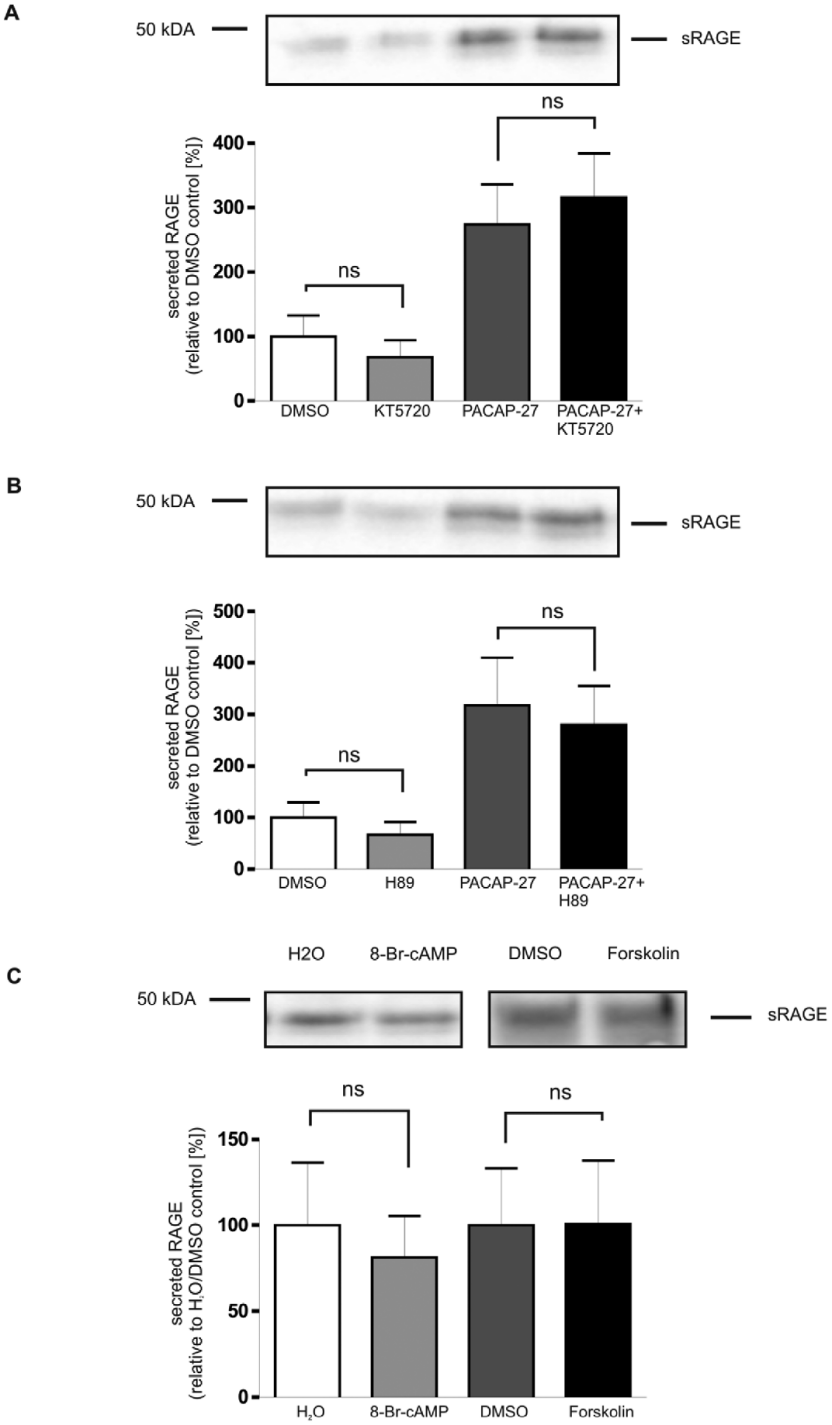
Analysis of adenylate cyclase/protein kinase A (PKA) signaling on the PACAP-induced RAGE secretion. (A) and (B), effect of protein kinase A inhibition; (C) effect of adenylate cyclase/protein kinase A activation. (A) and (B), PAC1/RAGE cells were pre-treated for 1 h with either PKA inhibitor H89 (5 µM, figure part A) or KT5720 (1 µM, figure part B) then 300 nM PACAP-27 was added for 2 h. The effects of PKA inhibitors were also analyzed in the absence of PACAP stimulation. Secreted RAGE was detected and quantified as described in Figure 1. Shown are the mean effects ± S.D., significance was determined by the One-way ANOVA (ns  =  P>0.05). For quantification the experiments were performed in triplicates. (C) Cells were incubated with either adenylate cyclase stimulator Forskolin (50 µM) or PKA activator 8-Br-cAMP (1 µM) for 2 h. Secreted RAGE in the cell culture supernatant was detected and quantified by Western Blot as described above. H_2_O, DMSO: mock-treated cells (negative controls).

As the name PACAP implies, it activates the adenylate cyclase/protein kinase A (PKA) pathway. Therefore the effects of H89 (5 µM), a cell-permeable inhibitor of PKA, and KT5720 (1 µM), a more specific cell-permeable inhibitor of PKA, on the stimulation of RAGE shedding were tested. Both inhibitors had no effect on the PACAP-induced shedding of RAGE (Figure 9A, B).

We also analyzed the effect of direct adenylate cyclase/PKA activation on RAGE shedding. For this purpose RAGE expressing cells were treated with either Forskolin (50 µM), an activator of the adenylate cylcase, or 8-Bromoadenosine 3′, 5′-cyclic monophosphate (8-Br-cAMP, 1 µM), an activator of PKA. Both compounds did not activate the shedding of RAGE (Figure 9C) supporting the assumption that the adenylate cylase/PKA signaling cascade does not contribute to the regulated proteolysis of RAGE. As control, the biological activity of Forskolin was validated using the GloSensor cAMP assay (Promega), data not shown.

PAC1 receptor activation also leads to stimulation of phospholipase C (PLC) which produces the second messenger molecules DAG and IP3 (Inositol trisphosphate) [23]. Afterwards calcium is released from intracellular stores leading to activation of protein kinase C (PKC).

**Figure 10:**
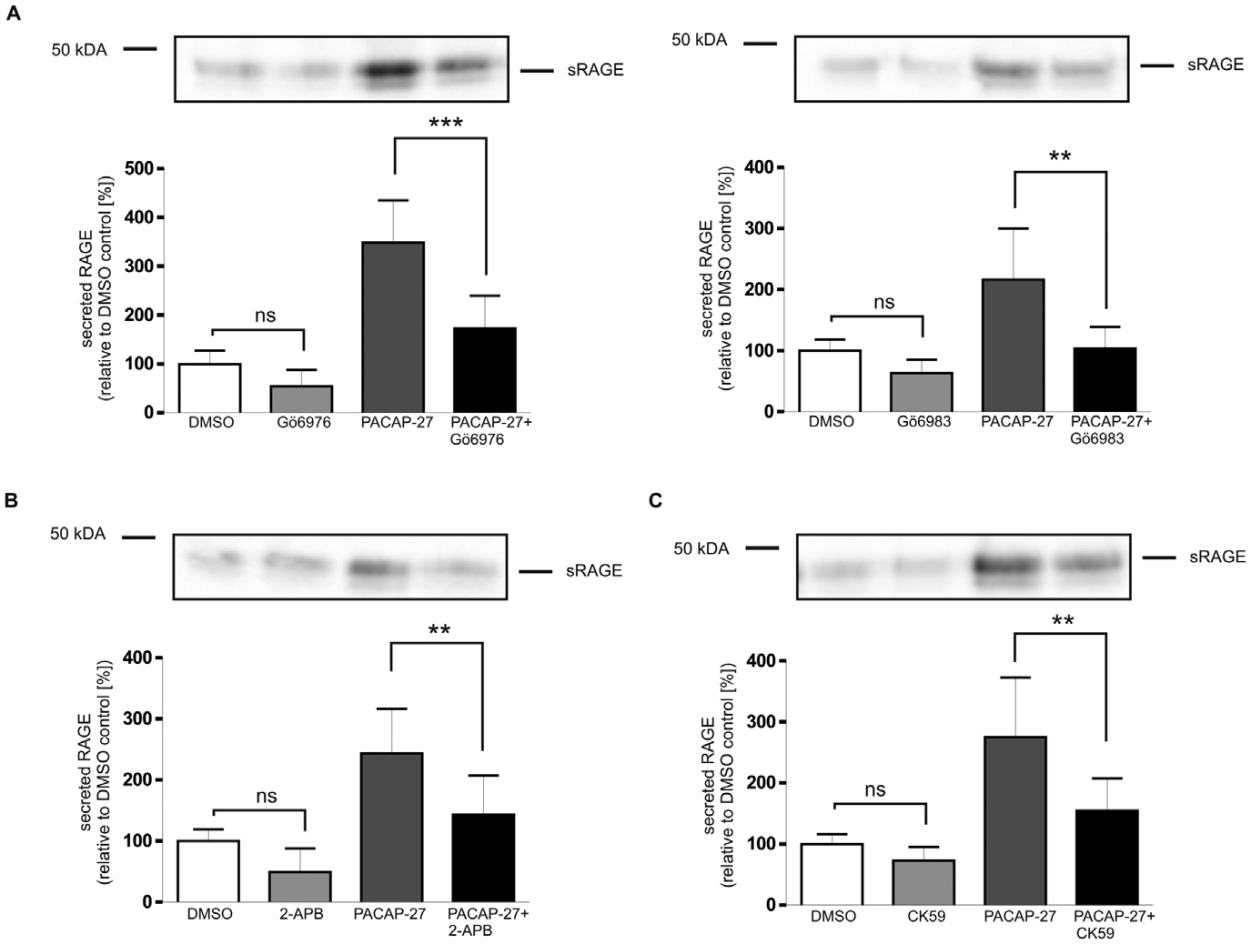
Effect of Ca^2+^ on the shedding of RAGE. PAC1/RAGE cells were pre-incubated for 1 h with respective inhibitor and then 300 nM PACAP-27 was added for 2 h. Secreted RAGE in the cell culture supernatant was detected and quantified as described in Figure 1. Shown are the mean effects ± S.D., significance was determined by the One-way ANOVA test (ns = P>0.05; **  =  P<0.01; ***  =  P<0.001). (A) Effect of protein kinase C inhibitors Gö6976 and Gö6983 (both 1 µM); (B) effect of the IP3 receptor modulator 2-APB (75 µM), and (C) effect of the calcium/calmodulin-dependent protein kinase II inhibitor CK59 (20 µM). In all experiments effects of inhibitors were also analyzed in the absence of PACAP stimulation.

For analysis of PACAP-induced activation of different PKC isoenzymes, two different PKC inhibitors were applied: Gö6976 selectively inhibits calcium-dependent PKC isoforms (PKCα, PKCβI); Gö6983 selectively inhibits the PKC isozymes PKCα, PKCβ, PKCγ, PKCδ and PKCζ but it does not block PKCµ (IC_50_ = 20 mM). Both PKC inhibitors reduced the PACAP-induced shedding of RAGE: Treatment with Gö6976 and Gö6983 (both 1 µM) resulted in an approximate 60% and 50% inhibition of PACAP-stimulated RAGE shedding (Figure 10A). For further analysis of the contribution of Ca^2+^ in the shedding of RAGE, the PAC1/RAGE cells were pre-treated with the IP3 receptor antagonist 2-APB (75 µM) and then the shedding process was induced for 2 h with PACAP-27. Compared to mock-treated cells, 2-APB decreased the PACAP-induced release of soluble RAGE about 40% (Figure 10B).

Besides PKC, Ca^2+^ also activates calmodulin dependent protein kinase II (CaMKII) and PACAP-initiated stimulation of CaMKII has been described [30]. Treatment of PAC1/RAGE cells with CK59 (20 µM), a cell-permeable CaMKII inhibitor (IC50<10 µM), decreased the PACAP-induced shedding of RAGE about 43% (Figure 10C). These results demonstrate that Ca^2+^ signaling, PKCα/PKCβI and CaMKII play substantial roles in the activation of metalloproteinase-mediated RAGE shedding.

**Figure 11:**
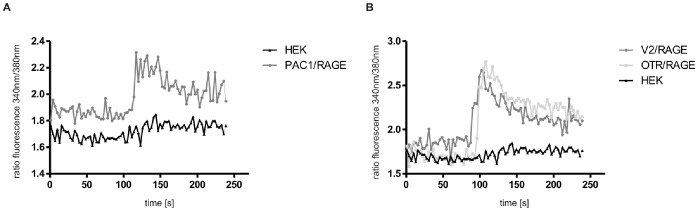
Measurement of intracellular calcium signaling. PAC1/RAGE (A), V2R/RAGE and OTR/RAGE cells (B) as well as HEK cells (A/B) were incubated for 30 min with 1,5 µM fura 2-AM. Then the cells were suspended and collected by centrifugation (0.4 g), after suspending cells in calcium buffer, measurements were performed with a spectrofluorometer. The respective agonists were applied to the cells after 100 s. As a negative control, either PACAP-27 or OT and AVP were added to HEK cells. Rising intracellular Ca^2+^ levels are detected by an increase in the fluorescence ratio 340 nm/380 nm.

Since Ca^2+^ appears to be a dominant regulator of induced RAGE shedding, we analyzed whether agonistic activation of our cell lines leads to elevated intracellular Ca^2+^ levels. Depending on the coexpressed GPCR, RAGE expressing cells were treated with either PACAP-27, AVP or OT (each 300 nM). In all RAGE/GPCR coexpressing cells agonist treatment induced a strong intracellular Ca^2+^ signal as measured via calcium sensor Fura-2-AM (Figure 11). In HEK cells neither hormone induced calcium signaling. Thus activation of GPCRs is required for elevation of intracellular calcium levels. The finding that also activation of the V2 receptor can modulate intracellular calcium is an explanation for its involvement in induced RAGE shedding, because by using PKA inhibitor KT5720 we could exclude an involvement of cAMP/PKA signaling in V2R-induced RAGE shedding (data not shown).

**Figure 12:**
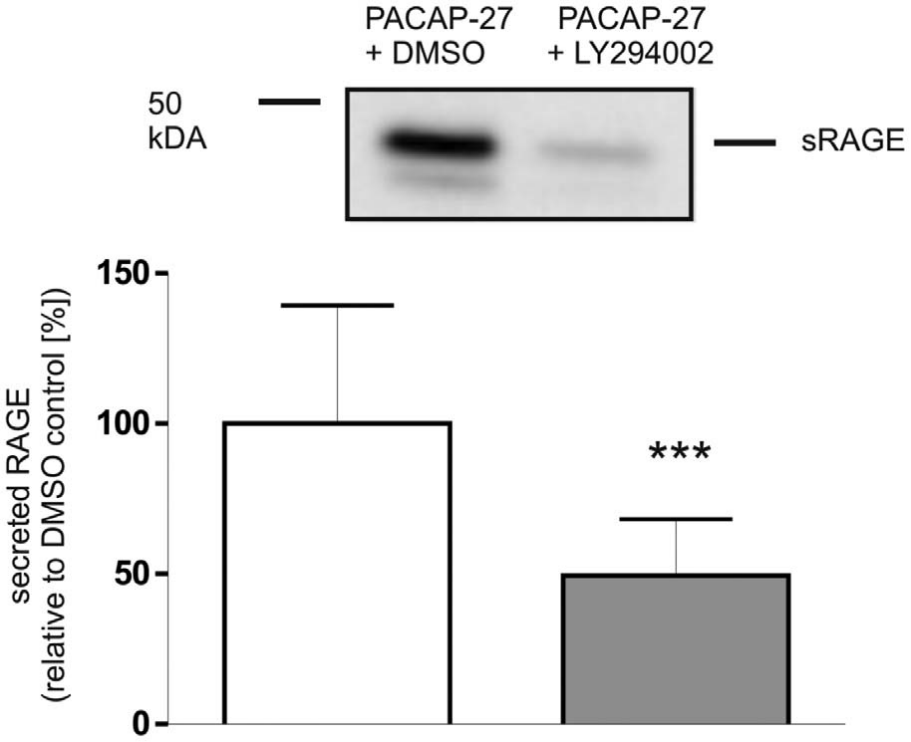
Role of phosphatidylinositol 3-kinase in PACAP-induced RAGE shedding. PAC1/RAGE cells were pre-incubated for 1 h with PI3K inhibitor LY294002 (50 µM) and then 300 nM PACAP-27 was added for 2 h; DMSO: mock-treated cells (negative control). Secreted RAGE in the cell culture supernatant was detected and quantified as described in Figure 1. Shown are the mean effects ± S.D., significance was determined by the Student’s unpaired *t* test (***  =  P<0.001).

**Figure 13:**
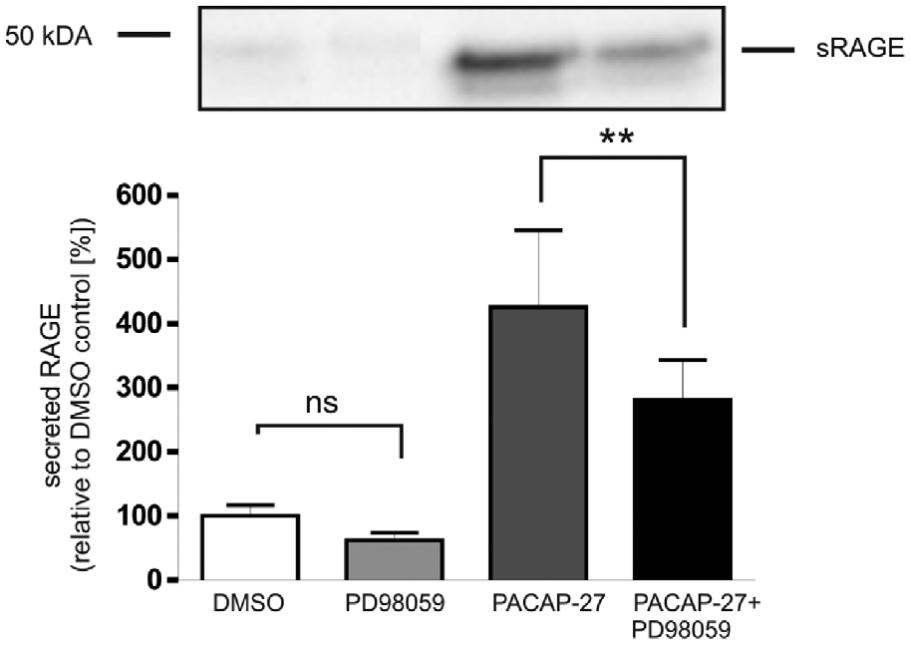
Role of the MEK1 MAP kinase pathway in PACAP-induced RAGE shedding. PAC1/RAGE cells were pre-incubated for 1 h with MEK1 Inhibitor PD98059 (50 µM) and then 300 nM PACAP-27 was added for 2 h. Secreted RAGE was detected and quantified as described in Figure 1. Shown are the mean effects ± S.D., significance was determined by the One-way ANOVA test (ns = P>0.05; **  =  P<0.01).

Since PACAP is also able to activate the phosphatidylinositol 3-kinase (PI3-kinase) [31] and mitogen-activated protein kinases (MAP kinases) [32], [33], we also investigated whether these signaling pathways contribute to the PACAP-induced shedding of RAGE. Treatment of PAC1/RAGE cells with 50 µM LY294002 (a specific, cell-permeable inhibitor of PI3-kinase) decreased PACAP-induced shedding of RAGE about 50% (Figure 12). The use of PD98059 (50 µM), which is a selective, reversible and cell-permeable inhibitor of MAP kinases MEK1 and MEK2, decreased PACAP-induced shedding of RAGE about 34% (Figure 13).

**Figure 14:**
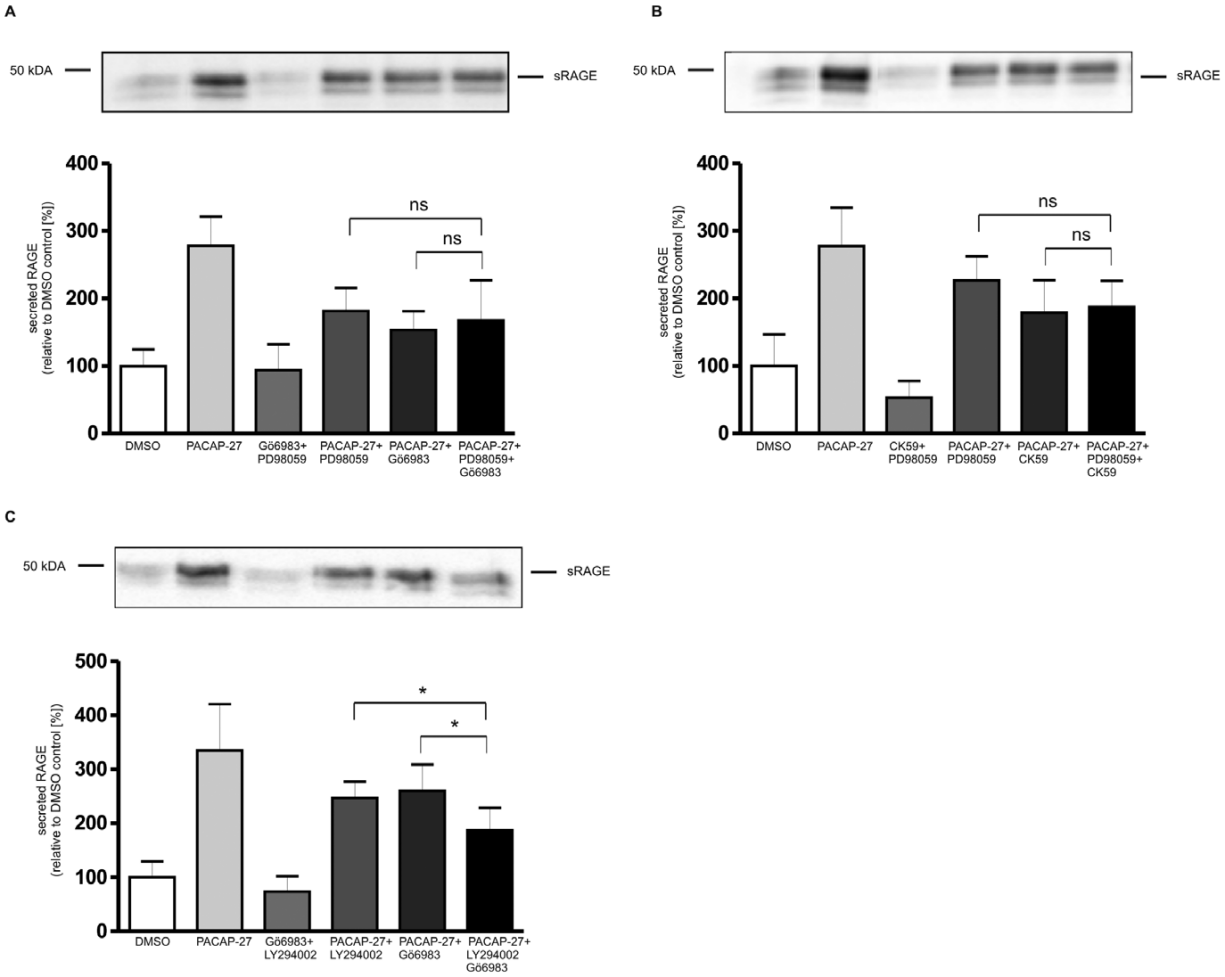
Influence of simultaneously added kinase inhibitors on the shedding of RAGE. PAC1/RAGE cells were pre-incubated for 1 h with (A) MEK1 inhibitor PD98059 (50 µM) and PKC inhibitor Gö6983 (1 µM); (B) MEK1 inhibitor PD98059 (50 µM) and CaMKII inhibitor CK59 (20 µM) or (C) PI3K inhibitor LY294002 (50 µM) and PKC inhibitor Gö6983 (1 µM). Then 300 nM PACAP-27 was added for 2 h. Secreted RAGE was detected and quantified as described in Figure 1. Shown are the mean effects ± S.D., significance was determined by the One-way ANOVA test (ns = P>0.05; *  =  P<0.05).

**Figure 15:**
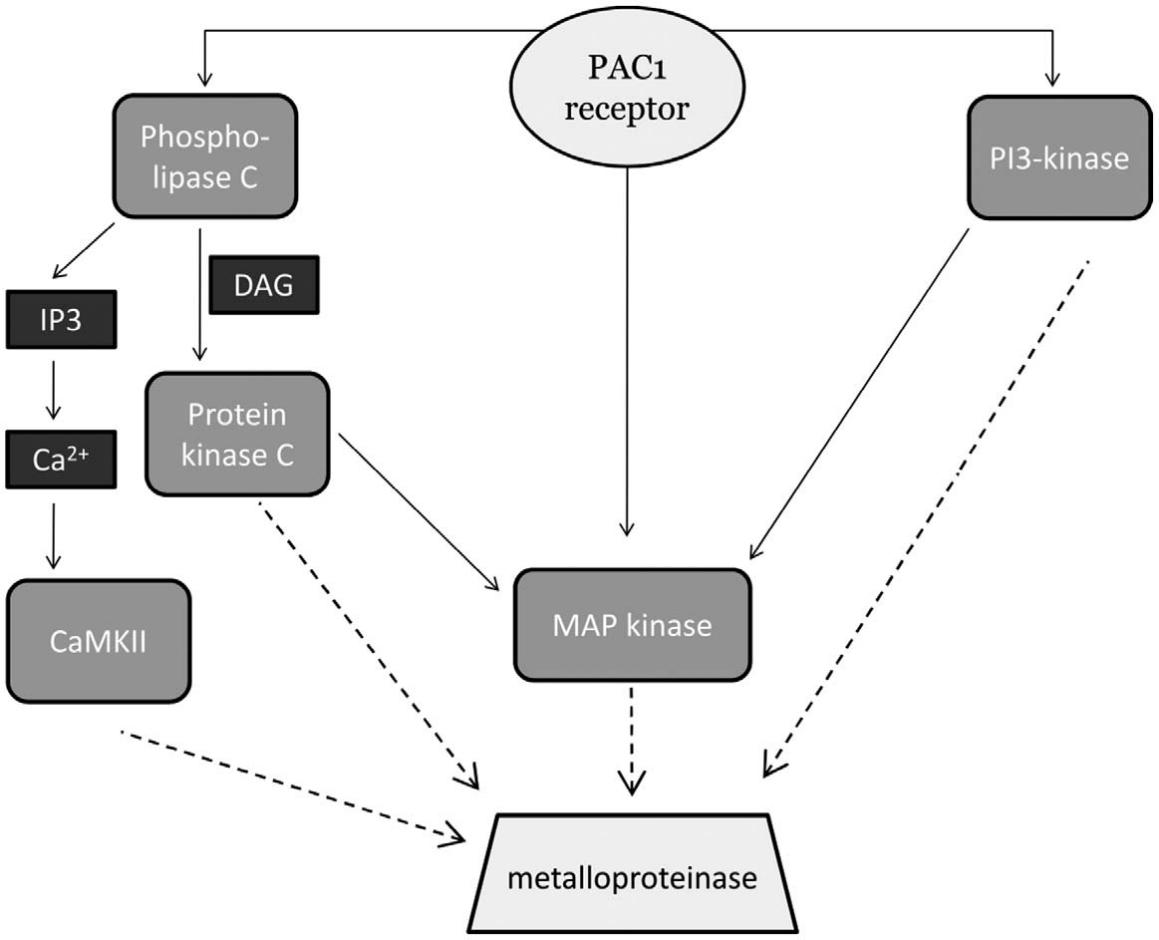
Model of the intracellular pathways involved in PACAP-induced RAGE shedding. In this study we demonstrated an involvement of MAP kinase, PI3-kinase, PKC and CaMKII in PAC1 receptor-induced activation of metalloproteinase-mediated RAGE shedding. Molecular mechanisms linking signaling cascades with metalloproteinase activation are still unknown (dotted lines). CaMKII: calmodulin-dependent protein kinase II; DAG: diacylglycerol; IP3: inositol 1,4,5-triphosphate; MAP kinase: mitogen-activated protein kinase; PI3-kinase: phosphatidylinositol 3-kinase.

By using various inhibitors we identified several signaling pathways contributing to RAGE shedding. Next we analyzed whether these signaling pathways are contributing in parallel to the shedding of RAGE. Combinatorial treatment of cells with MAP kinase inhibitor and either PKC or CamKII inhibitor did not result in an enhanced inhibition of PACAP-induced RAGE shedding (Figure 14). When compared to the presence of only one inhibitor, parallel inhibition of PI3 kinase and PKC resulted in reduced shedding of RAGE. The latter result suggests that activation of metalloproteinase-mediated RAGE shedding via PI3K and PKC is mediated by signaling pathways acting in parallel. CamKII and PKC appear to rather contribute to cascade-like signaling which also involves MAP kinases (Figure 15).

### Analysis of RAGE Shedding in Mouse Lungs

**Figure 16:**
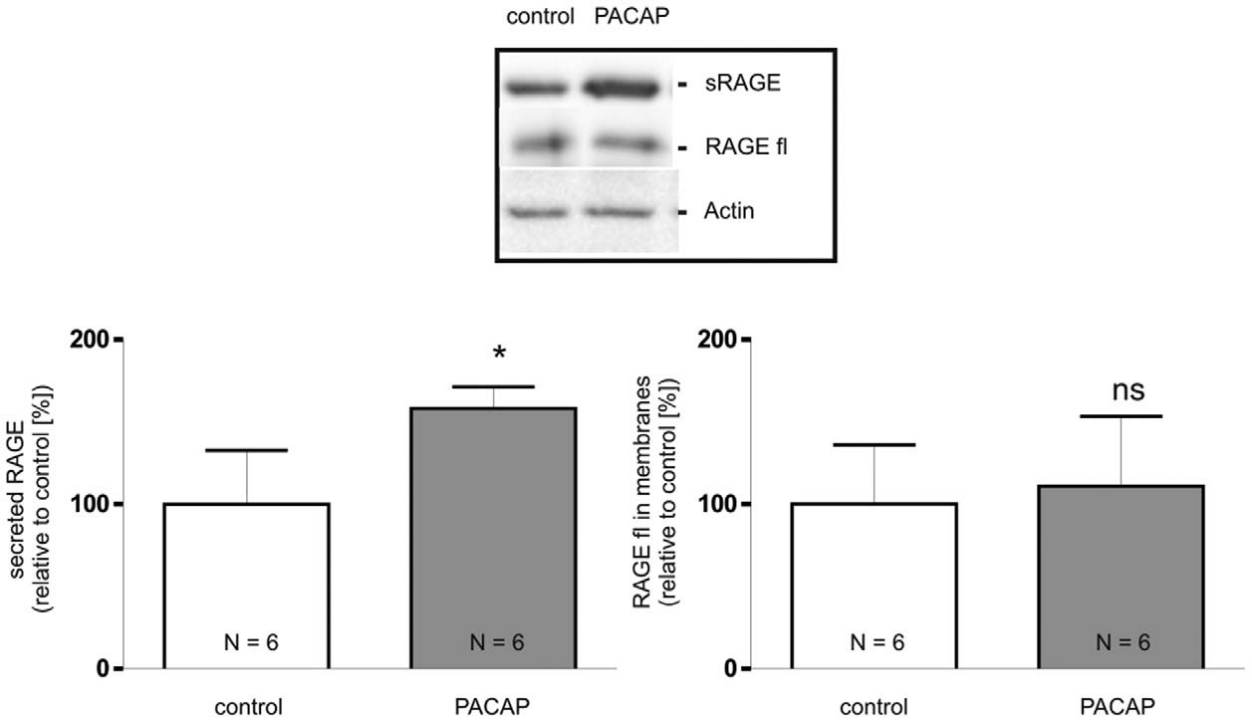
Analysis of PACAP-induced RAGE shedding *in vivo*. Mices (N = 6) were treated intranasally with PACAP-38 for 3 months. Afterwards mouse lungs were dissected and membrane proteins and soluble proteins were separated following tissue homogenization by ultracentrifugation. Secreted RAGE (sRAGE) and full-length RAGE (RAGE fl) were detected by Western blotting using an antibody directed against mouse RAGE (MAB1179) followed by an anti-rat antibody labeled with horse radish peroxidase, as loading control actin was detected. Shown are the mean effects ± S.D., significance was determined by the Student’s unpaired *t* test (ns  =  P>0.05; *  =  P<0.05).

Finally we were interested whether PACAP-stimulated shedding is also evident *in vivo*. For this purpose, mice were treated for 3 months intranasally with either PACAP-38 or mock administration solution [24]. Since highest amounts of RAGE are found in the lung and PACAP receptors are also present in this organ, mouse lungs were isolated and fractionated into portions containing either soluble lung proteins or membrane proteins. In the fraction of soluble lung proteins the amount of sRAGE was increased by about 60% in PACAP-treated mice (Figure 16). The amount of membrane-bound full-length RAGE was not altered by PACAP-treatment (Figure 16).

## Discussion

In this report we provide evidence that metalloproteinase-mediated shedding of the type I membrane protein RAGE is inducible by activation of G protein-coupled receptors which are expected to be linked to different main signal transduction pathways. We demonstrate that RAGE shedding can be induced via activation of the V2 vasopressin, the oxytocin and the PAC1 receptor. The shedding of RAGE was induced 2 to 5-fold after activation of examined GPCR. Observed differences provide no information about the efficiency of individual GPCR-activated shedding induction, because cell lines expressing different amounts of GPCR and RAGE were compared. Nevertheless, our results clearly demonstrate that the RAGE shedding machinery is inducible via activation of various GPCRs. For the detailed analysis of involved signaling pathways we focused on the multi-branched signaling network of the PAC1 receptor since this also covers the more restricted signaling routes of V2R and OTR.

By a biotinylation experiment, we demonstrated that PACAP-induced RAGE shedding takes place at the cell surface where a substantial amount of plasma membrane-located RAGE is proteolytically cleaved after activation of PAC1 receptors. Similar results have been published for PMA-induced RAGE shedding [16]. A detailed analysis for the PAC1 receptor demonstrated that Ca^2+^ signaling, PKCα/PKCβI, CaMKII, PI3 kinase and MAP kinases are involved in the shedding activation process. A contribution of adenylate cyclase/PKA signaling in PACAP-induced RAGE proteolysis was not evident in our cellular system. In V2R/RAGE cells we could also not detect an involvement of adenylate cyclase/PKA signaling in AVP-induced RAGE shedding. In all our cell lines expressing different GPCRs (PAC1/RAGE, V2R/RAGE and OTR/RAGE) we observed agonist-induced intracellular calcium signaling. Therefore we conclude that Ca^2+^ seems to be an important regulator of the RAGE shedding machinery.

Analysis of lungs isolated from PACAP-treated mice further showed that the shedding of RAGE is also inducible *in vivo*. So far induced shedding of RAGE was demonstrated only for unphysiological stimulators such as the phorbolester PMA and the calcium ionophore A23187 [16].

In connection with the development of Alzheimeŕs disease it is important to consider that at least two aspects of metalloproteinase-mediated shedding processes are essential: (i) cleavage of APP by ADAM10 prevents generation of neurotoxic Aβ peptides; (ii) cleavage of full-length RAGE by ADAM10 prevents binding of the RAGE ligand Aβ to cell-bound RAGE [34]. Cell-bound RAGE is known being an Aβ transporter importing Aβ from the blood into the brain and also being a neuronal receptor mediating neurotoxic effects of Aβ. Thus activation of ADAM10-mediated cleavage of RAGE and APP should counteract the development of AD, and furthermore stimulation of RAGE shedding should prevent at least some pro-inflammatory effects associated with diabetes mellitus. The therapeutic potential of PACAP in the treatment of AD was investigated by us in an AD mouse model. Intranasal application of PACAP-38 increased α-secretase-mediated APP processing, induced expression of various neuroprotective genes and improved memory in mice [24]. In the same study, we detected a strong reduction of the RAGE mRNA level in the brain of PACAP-treated mice. It is known that RAGE expression is induced by an autocrine mechanism of ligand binding and RAGE signaling [7]. This activation loop can be disrupted by the shedding of membrane-bound signaling capable RAGE and/or by decreasing the amount of RAGE ligands such as Aβ. In our mouse model both options are likewise possible. This finding and our current data demonstrate that shedding of at least APP and RAGE is inducible by administration of PACAP *in vivo*. Thus, application of PACAP may also have therapeutic potential for the treatment of AD or inflammatory diabetic conditions in humans.

In cells coexpressing RAGE and the PAC1 receptor, the shedding of RAGE was inducible by various PAC1 receptor agonists. Induction of RAGE shedding was dose-dependent and saturable. A similar effect has been observed for PACAP-induced shedding of APP [19] suggesting activation of the same shedding-machinery. The broad spectrum metalloproteinase inhibitor GM6001 was able to prevent the PACAP-induced shedding of RAGE. ADAM10 and ADAM17 are the main sheddases responsible for ectodomain shedding of a number of cell surface proteins. However, in a previous study we found ADAM10 and MMP9 to mediate RAGE shedding [16]. The contribution of these proteinases in PACAP-induced RAGE shedding was analyzed in more detail by using the ADAM10/MMP9-selective inhibitor GI254023X. In our cellular assay 25 µM and even a concentration of 1 µM GI254023X strongly reduced constitutive RAGE shedding; also PACAP-induced shedding of RAGE was significantly reduced. At a concentration of 100 nM, a slight inhibition of RAGE shedding was still observed. In *in vitro* assays with recombinant proteinases, GI254023X discriminates between ADAM17 (IC_50_ = 541 nM) and ADAM10 (IC_50_ = 5.3 nM)/MMP9 (IC_50_ = 2.5 nM) [28]. However, in cellular assays, IC_50_ values appear to be much higher: at 30 µM GI254023X has been reported to reduced PMA-induced shedding by only 20% [28], [29]. In the case of ADAM10-mediated CD23 shedding, 1 µM of GI254023X was sufficient to block about 75% of CD23 release [35]. Thus, our observation that the constitutive as well as the PACAP-induced shedding of RAGE was inhibited with 1 µM GI254023X about 67% and 75% strongly argues for a contribution of ADAM10 in that process. By applying siRNA-mediated knock-down experiments we could confirm the role of ADAM10 and MMP9 in constitutive and PACAP-induced RAGE shedding and in addition a contribution of ADAM17 in PACAP-induced RAGE shedding was observed. According to our experimental setting, we cannot exclude the contribution of other proteinases in RAGE shedding. However, the involvement of ADAM10 in constitutive RAGE shedding is supported by findings of other research groups where either experiments with genetically modified mouse embryonic fibroblasts or an ADAM10 overexpression strategy were utilized [14], [15].

Our comparative analysis of PACAP-induced RAGE shedding versus phorbol ester-induced shedding revealed a similar kinetic in the examined cellular system. Shedding increased continuously during the first three hours after its induction and reached a maximum after about four hours. Similar results for PMA-induced shedding have been described [36]. The potency of GPCR-mediated cellular activation and hence the induction of ectodomain shedding depends on the GPCR density on the cell surface and on the agonist concentration. Our finding that intranasal application of PACAP to mice is sufficient for induction of RAGE shedding in the mouse lung demonstrates that an increased shedding of RAGE is achievable by activation of endogenously present PACAP receptors. Large amounts of full-length RAGE are expressed in the lung [1]. Therefore the lung can possibly serve as a pool for the release of soluble RAGE into the blood stream upon shedding induction. As already mentioned, soluble RAGE prevents proinflammatory full-length RAGE signaling.

The name PACAP (*Pituitary adenylate cyclase-activating polypeptide*) implies adenylate cyclase and subsequently protein kinase A (PKA) as targets of PACAP receptor activation. However, in our study we did not observe any effects of either adenylate cyclase/PKA activation or inhibition on the shedding of RAGE. PACAP-induced APP processing was also identified to occur independently from the adenylate cyclase/PKA system [19]. ADAM-mediated shedding processes seem to be largely independent from adenylate cyclase/PKA signaling. Instead, calcium signaling is playing an important role. Our finding that AVP-induced activation of the vasopressin V2 receptor also induced RAGE shedding independent from adenylate cyclase/PKA signaling, can be explained by the fact that besides activation of adenylate cyclase/PKA signaling, intracellular calcium levels are also increased upon V2 receptor activation [37]. We observed a reduction of PACAP-induced RAGE shedding after treatment of cells with various inhibitors interfering with Ca^2+^ signaling. Inhibition of intracellular calcium release as well as blockade of calcium-dependent PKC isoforms or calcium/calmodulin-dependent protein kinase II (CaMKII) significantly reduced the PACAP-induced RAGE shedding. The importance of Ca^2+^ signaling in the induction of RAGE shedding is supported by our finding that activation of the oxytocin receptor leads to elevated sRAGE levels; this receptor is known to be connected to Ca^2+^ signaling [22].

A role for the phosphatidylinositol 3-kinase (PI3-kinase) pathway has been demonstrated for the anti-apoptotic effect of PACAP [31]. Therefore, we analyzed whether PI3-kinase is also involved in PACAP-mediated RAGE proteolysis. Inhibition of PI3-kinase using LY294002 strongly reduced the PACAP-induced RAGE shedding. We also identified MAP kinase signaling to contribute to PACAP-induced RAGE shedding. MAP kinases play important roles in the signal transduction of receptor tyrosine kinases (RTKs) such as the insulin receptor. Moreover, insulin signaling has been demonstrated to induce ADAM10-mediated shedding of Klotho [38]. Therefore, it is likely that also the ADAM10-mediated cleavage of APP and RAGE is inducible via activation of insulin receptors; for APP processing this has already been demonstrated [39], [40].

Given that increased Ca^2+^ levels are sufficient for induction of ADAM-mediated shedding processes, the activation of RAGE ectodomain shedding should be achievable via stimulation of different receptor classes including GPCRs, RTKs and ligand-controlled Ca^2+^ channels.

It is well documented that shedding processes can be induced by quite distinct stimuli. For example, the catalytic activity of ADAM17, but not that of ADAM10, can rapidly and reversibly be induced by signaling pathways stimulated by thrombin, EGF, lysophosphatidic acid and TNFα. Activation of ADAM17 was independent from its cytoplasmic tail but required the presence of the transmembrane domain and led to the exposure of the ADAM17 catalytic site [41]. It is elusive how these observations are linked on the molecular level, but they rather argue against a direct inside-out signaling mechanism. On the other hand, the C-terminus of ADAM10 is required for the induction of ADAM10 activity by calcium [42] and a putative PKC phosphorylation site and SH3 domains in the ADAM10 C-terminus are possibly involved in shedding activation [43]. Collectively viewed, the molecular mechanisms linking GPCR activation and protein ectodomain shedding appear to be multilayered and they also seem to be different for specific proteinases.

Independent from the missing links of cell and sheddase activation, our presented data provide evidence that pharmacological stimulation of RAGE shedding may open alternative treatment strategies for AD or RAGE-mediated diabetic complications in the future.

## Materials and Methods

### Antibodies and Reagents

The following antibodies were used: anti human RAGE N-terminal antibody Mab5328 (Millipore) detecting membrane-bound RAGE and rabbit serum Ab3260 detecting secreted RAGE [16]. For detection of RAGE in mouse samples, the antibody MAB1179 from R&D Systems was used. This antibody recognizes the extracellular domain of murine RAGE. The anti-actin antibody (A2066) was from Sigma-Aldrich. Anti ADAM10 (AB19026) and ADAM17 (AB19027) antibodies directed against cytosolic ADAM domains were from Millipore.

Secondary anti-rabbit and anti-mouse peroxidase-coupled antibodies and the ECL detection reagent were from Pierce (Rockford, USA). The anti-rat POD-coupled secondary antibody was from Merck (Darmstadt). All peptide hormones were from Bachem. All other chemicals were either from Merck Biosciences or Sigma-Aldrich.

### Expression Vectors

RAGE: The untagged wild-type human RAGE expression vector pcDNA6-RAGE was generated by using the human RAGE cDNA, including its own translation termination codon. The human RAGE cDNA was subcloned from clone IRALp962E1737Q2 into EcoRI/XbaI sites of pcDNA6/V5-HisB. PAC1: The rat PACAP type I receptor, which has 92% amino acid identity compared to the human receptor, was used in the experiments. The PAC1 coding region was fused at the 5′end to a myc epitope coding sequence and was combined at the 3′end to an oligonucleotide adaptor coding for a Rho epitope in a pcDNA3 vector. Expression plasmids for the C-terminal HA-tagged bovine V2 vasopressin and human oxytocin receptor carrying a N-terminal c-myc and FLAG epitopes and a C-terminal GFP-tag are described elsewhere [44], [45]. The HA-tagged bovine V2 vasopressin receptor cDNA sequence was subsequently subcloned into plasmid pcDNA3 (Invitrogen) generating pcDNA3-V2R-HA.

### Transfection of Cells

HEK Flp-In™ 293 cells (human embryonic kidney cells) (Invitrogen) were transfected with pcDNA3-PAC1 expression plasmid using Lipofectamine 2000 (Invitrogen) and selected via G418 to generate a stable cell line. Then one cell clone expressing the PAC1 receptor was transfected with pcDNA6-RAGE expression plasmid using Lipofectamine 2000 and selected via Blasticidin.

HEK 293 cells (ATCC) stably expressing the oxytocin receptor (OTR) [45] were transfected with the RAGE expression vector pcDNA6-RAGE to generate stably OTR/RAGE coexpressing cells. A single cell clone expressing RAGE and OTR was isolated via G418 and Blasticidin selection.

HEK 293 cells (ATCC) stably expressing the bovine vasopressin receptor (V2R) were generated by us by transfection of HEK 293 cells using plasmid pcDNA3-V2R-HA. A V2 vasopressin receptor-expressing cell clone was isolated by using G418 selection. Subsequently, this cell clone was transfected with plasmid pcDNA6-RAGE and a RAGE/V2R coexpressing cell clone was isolated via G418 and Blasticidin selection.

### Small Interference RNA Experiments

Stealth RNAi duplexes were purchased from Invitrogen, and transfections were performed according the manufacturer’s protocol. The RAGE cleavage assay was performed 48 h after transfection.

### Measurement of Intracellular Free Calcium Ion Concentration [Ca2+]i

Cells were incubated with 1,5 µM fura 2-AM in DMEM for 30 min at 37°C. Afterwards they were detached by using PBS/0,5 mM EDTA and centrifuged at 100 g for 5 min. Pellets were washed and resuspended in calcium buffer (10 mM HEPES, pH 7.4, 140 mM NaCl, 5 mM KCl, 0,5 mM MgCl_2_, 1,5 mM CaCl_2_, 10 mM glucose). Aliquots of the suspension were added to calcium buffer and transferred into a cuvette that was placed into a thermostatically controlled (37°C) holder. The agonists (final 300 nM) were applied to the cells after 100 s, and the changes of the [Ca2+]_i_ were monitored spectrofluorimetrically (Photon Technology Int., Birmingham (USA)).

The emission wavelength was set at 510 nm, and dual-wavelength excitations were performed at 340 and 380 nm. The change of the Ca^2+^ concentration was calculated by using the ratio 340 nm/380 nm.

### RAGE Cleavage Assays and Inhibitor Treatments

Cells coexpressing RAGE and GPCRs were seeded onto poly-L-lysine-coated 6 well plates and grown for 24 h to 80-90% confluence. Cells were washed twice with serum-free DMEM and then secretion medium (serum-free DMEM supplemented with 2 mM glutamine) was added. Experiments in the presence of inhibitors were performed by preincubating cells with the inhibitor in secretion medium for 1 h at 37°C and then PACAP hormones, arginine-vasopressin or oxytocin were added. After the appropriate incubation time, cell culture supernatants were collected centrifuged for 10 min at 660 g and proteins were precipitated with 10% trichloroacetic acid at 4°C. Proteins were analyzed by Western blotting. For detection of cellular proteins, the adherent cells on the 6 well plates were washed with PBS, then dissolved in reducing Laemmli buffer and assayed by Western blot analysis. For comparative and quantitative analysis, effects observed with solvent-treated cells were used as control and were set to 100%. As a further control, the effect of the inhibitors on RAGE shedding was also analyzed in the absence of hormones.

### Biotinylation of Cell Surface Proteins

Adherent cells were washed twice with PBS and then incubated for protein biotinylation with PBS containing 0.1 mM Sulfo-NHS-LC-Biotin (Pierce Biotechnology) for 30 min at room temperature. After washing with Tris-buffered saline (pH 7.4) the cells were incubated in secretion medium for 2 hours. The cells and supernatants were collected separately, and the supernatants were adjusted to 0.1% SDS. The cells were dissolved in 5% SDS, and then SDS was diluted to 0.1% by adding PBS. Biotinylated proteins were captured with NeutrAvidin Biotin-binding agarose (Pierce) for 2 h at 4°C. After that, the agarose beads were washed three times with PBS/0.1% SDS. Proteins were eluted from the binding agarose with reducing Laemmli buffer and analyzed by Western blotting. Actin could not be detected in isolated biotinylated protein fractions, thus demonstrating the integrity of the cells during the course of the experiment. As a control, actin could be detected in the cell lysate (5% SDS fraction; see above).

### Western Blotting

Proteins of the cell culture supernatant or cell lysates were separated by 10% SDS-PAGE and blotted to nitrocellulose membranes (GE Healthcare). Membranes were probed with the appropriate primary and secondary antibodies and the labeled proteins were detected by chemiluminescence using the VersaDoc system (Bio-Rad Laboratories, Munich, Germany) and quantified via the AIDA 4.25 software (Raytest, Straubenhardt).

### Gelatin Zymography

Cells were washed twice with serum free DMEM, then serum free DMEM was added to the cells. After 18 h of incubation at 37°C, supernatants were collected and cleared from cells by centrifugation (660×g, 10 min). For gelatin zymography 30 µl of serum-free cell culture supernatant was mixed with 10 µl of 4× NuPAGE LDS Sample Buffer (Invitrogen), and subsequently proteins were separated under non-reducing conditions in 10% SDS-polyacrylamide gels containing 0.1% gelatin. After electrophoresis gels were washed in 2.5% Triton X-100 at room temperature for 1 h and then incubated for 16 h at 37°C in reaction buffer (100 mM Tris-HCl, pH 7.5, 10 mM CaCl_2_). Finally the gels were stained with Coomassie Blue. Clear areas within the stained gel result from digestion of gelatin by the gelatinase activity of either MMP9 or MMP2.

### Treatment of Mice and Samples Preparation

#### Ethics Statement

All animal experiments performed comply with the current German laws. Approval of animal studies by: Landesuntersuchungsamt Rheinland-Pfalz, Dienststelle Tierschutz, Mainzerstraße 112, 56068 Koblenz, Germany.

Approval ID: 177-07/051-17.

#### Treatment

Three-month-old male APP[V717I] mice were treated with PACAP38 for additional 3 months. The peptide solution (1 µg/µl in 0.5% chitosan glutamate and 0.5% NaCl in water; pH 4) was prepared as described [46]. The solution of PACAP38 was administered intranasal to APP[V717I] mice 5 days a week, for 3 months. The mice were treated with 10 µg PACAP38 per day, 10 µl for each mouse (5 µl/nostril); 5 µl/nostril of the inert carrier was given to the control group. Mice were sacrificed and then removed lungs were frozen on dry ice, and then stored at −80°C.

#### Samples preparation

Mouse lung samples were divided into pieces in liquid nitrogen and ice cold homogenization buffer (1,4 M NaCl/20 mM Tris/HCl pH 7,5 with 1 proteinase inhibitor tablet Complete Mini (Roche) per 10 ml) in the fourfold volume of the lung weight was added. Samples were homogenized in a tissue lyser (Qiagen, 30 Hz, 2×2 min) and the suspension was centrifuged for 2 h (100 000 g, 4°C), then the supernatant was transferred in a new tube. The pellets containing insoluble lung material were resuspended in the fourfold volume of homogenization buffer with the tissue lyser (30 Hz, 2×2 min) and afterwards centrifuged for 15 min (16 000 g, 4°C). Then the supernatant was discarded and the remaining pellet was suspended in a twofold volume of TBS (with proteinase inhibitor tablet Complete Mini) using the tissue lyser. This suspension was used for analysis of membrane proteins.

The protein contents of the soluble and membrane protein fractions were determined by the Bradford method. Proteins were analyzed by Western Blotting.
